# Gastric Varices in Absence of Splenic Vein Thrombosis: A Rare Entity of Idiopathic Non-Cirrhotic Portal Hypertension

**DOI:** 10.7759/cureus.1179

**Published:** 2017-04-19

**Authors:** Vivek Choksi, Binna Chokshi, Andrew Chu, Deepa Mandale, Daniel L Wolfson, Steven Kaplan, Hamid Feiz

**Affiliations:** 1 Internal Medicine Residency, Aventura Hospital and Medical Center; 2 Internal Medicine Department GME, Aventura Hospital and Medical Center; 3 Internal Medicine, Aventura Hospital and Medical Center; 4 Gastroenterology, Aventura Hospital and Medical Center; 5 Internal Medicine/Hospitalist Medicine, Aventura Hospital and Medical Center

**Keywords:** isolated gastric varices, non-cirrhotic portal hypertension, idiopathic

## Abstract

Idiopathic non-cirrhotic portal hypertension (INCPH) is portal hypertension (PHT) without cirrhosis and other identifiable causes. Esophageal and gastric varices are seen in INCPH which are mostly asymptomatic. We present a rare case of symptomatic isolated gastric varices (IGV) in the setting of INCPH.

We report a case of a 60-year-old man who presented with an acute onset of hematemesis and no identifiable history. Upon further evaluation, he was found to have non-bleeding dilated gastric varices on esophagogastroduodenoscopy (EGD) and PHT without cirrhosis. Our patient is unique because he has symptomatic IGV and INCPH.

## Introduction

The most common cause of portal hypertension (PHT) is cirrhosis; however, in the absence of cirrhosis, the condition is termed non-cirrhotic portal hypertension (NCPH). Pre-hepatic, intra-hepatic, and post-hepatic causes contribute to NCPH. When other causes of PHT are not identified, the term given to such a condition is idiopathic non-cirrhotic portal hypertension (INCPH).

There are multiple other terms used globally, and although there are reported differences in etiology, epidemiology, and hepatic pressures, these various presentations likely reflect the vast spectrum of the condition itself and not the distinct diseases making INCPH an umbrella term.

Even though there are several theories as to the cause of INCPH, the etiology is unknown. There is a higher incidence of this disease in socioeconomically disadvantaged patients who have poor living conditions and increased pathogen exposure [1]. INCPH has histological features of dilated sinusoids, phlebosclerosis, and dense portal fibrosis. The course is more benign than cirrhotic portal hypertension (CPH) because liver function is preserved. Management is primarily aimed at decreasing variceal bleeding.

INCPH can have esophageal and gastric varices. Gastric varices arising without the involvement of esophageal varices (EV) are termed isolated gastric varices (IGV) . Gastric varices occur in approximately 20% of the patients with PHT [2]. However, IGV is seen in 5% of cirrhotic patients and up to 10% of NCPH patients [3]. Gastric variceal bleeding develops only in 25% of the patients, yet, it has higher mortality than esophageal variceal bleeding [2].

## Case presentation

A 60-year-old-male with past medical history of hypertension, hyperlipidemia, and nephrolithiasis presented with acute onset of hematemesis. He denied history of non-steroidal, anti-inflammatory drugs or alcohol use. He was not on any anticoagulant or antiplatelet. Upon admission, his vital signs were: temperature 97.8°F, pulse 86 beats/minute, blood pressure 104/57 mmHg, respiratory rate 13/minute, pulse oximetry saturation 100% on room air.

On physical examination, he was not altered or in acute distress. The abdominal examination was significant for non-radiating epigastric tenderness.

**Figure 1:**
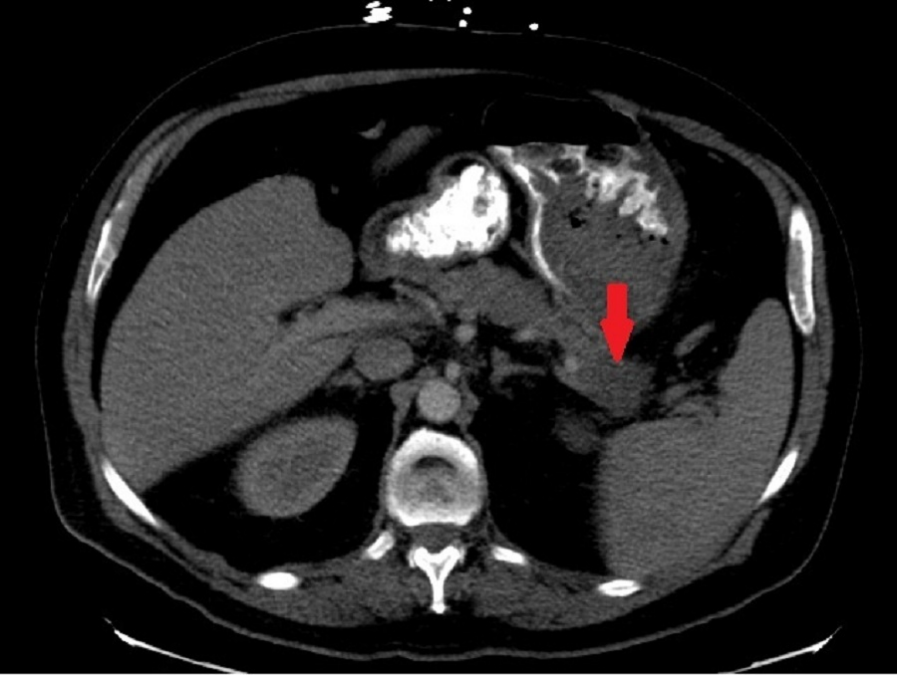
CT scan of the abdomen CT scan of the abdomen showing heterogenous mass (red arrow) within the tail of the pancreas.

On admission, his hemoglobin was 6.3 g/dL, hematocrit was 21%, and platelet count 264,000 u/L with minimally elevated blood urea nitrogen of 23 mg/dL. His coagulation panel, lipase and liver function tests were within normal limits. Viral hepatitis panel was negative. Computed tomography (CT) scan of the abdomen and pelvis with intravenous contrast showed a 2 cm area of low-density mass in the tail of the pancreas (Figure 1).

**Figure 2:**
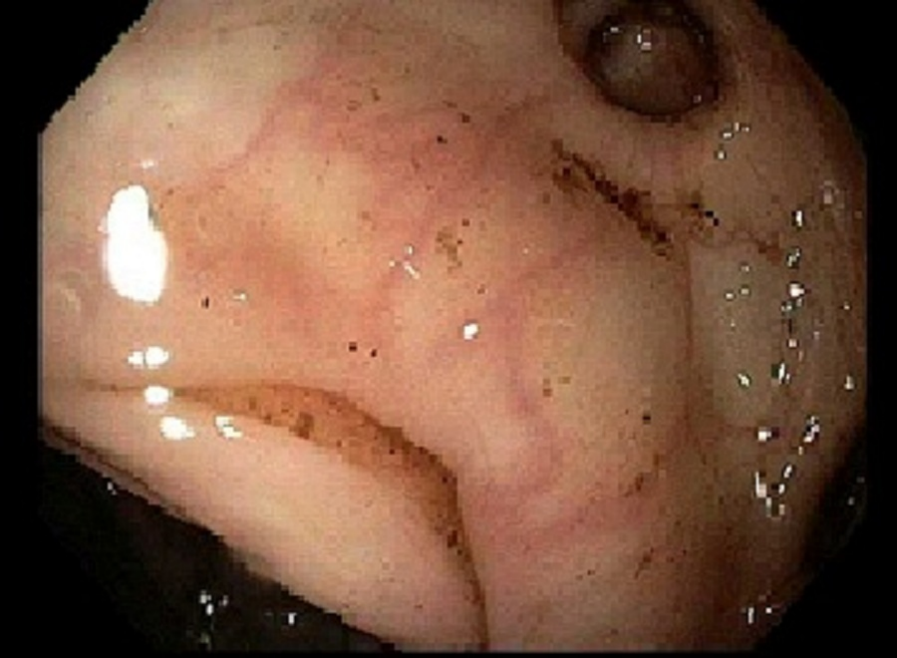
EGD of the stomach Esophagogastroduodenoscopy (EGD) image of the stomach body showing mild varices.

He was started on intravenous (IV) fluid resuscitation and IV pantoprazole and was transfused with four units of packed red blood cells. He went for emergent esophagogastroduodenoscopy (EGD) that showed non-bleeding dilated gastric varices (Figure 2).

Ultrasound of the portal venous system showed a patent portal vein and a splenic vein. Varices arising from porto-splenic confluence towards the left side of the abdomen were seen. A magnetic resonance imaging (MRI) scan of the abdomen showed hypovascular and partially necrotic 4 cm mass in the pancreatic tail. There was no occlusion of the surrounding vessels and no regional lymphadenopathy. A nuclear scan of the liver and spleen showed hepatosplenomegaly with mild colloid shift consistent with PHT.

**Figure 3:**
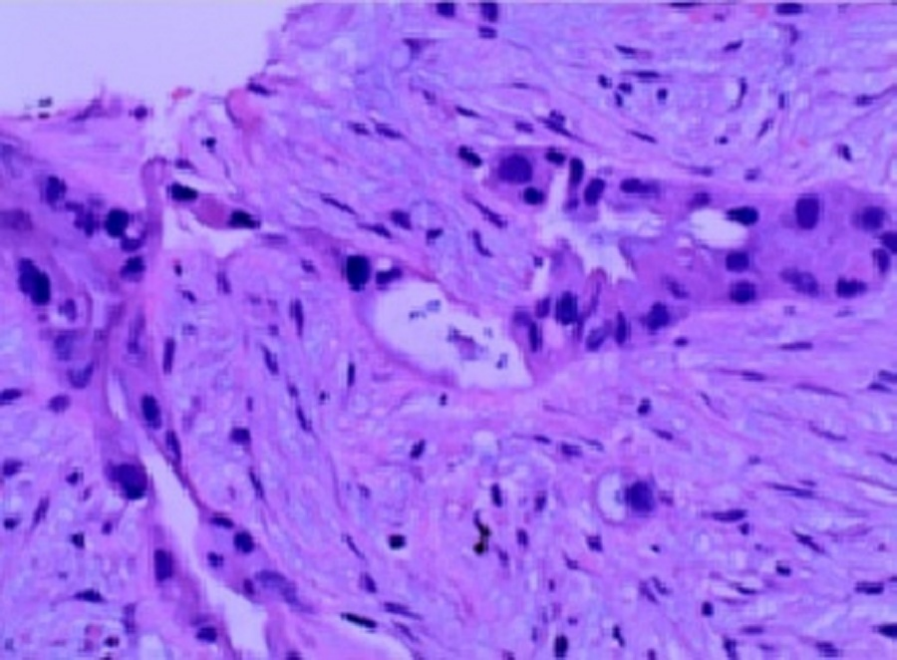
Histopathology of the pancreatic biopsy Pathology of the pancreatic tail biopsy showing malignant cells.

During the hospital course, the pancreatic mass was biopsied which turned out to be a moderately differentiated tubular adenocarcinoma (Figure 3).

**Figure 4:**
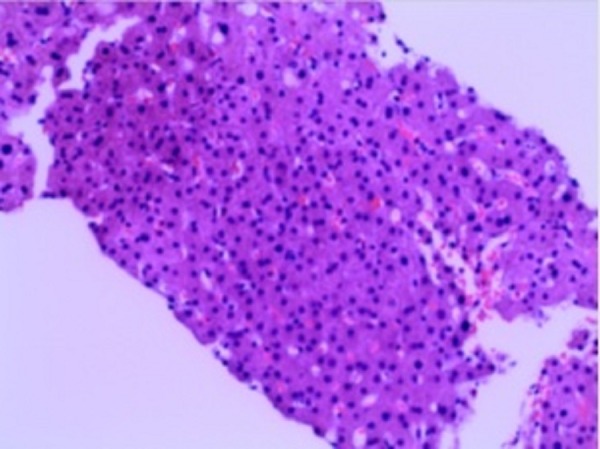
Histopathology of the liver Pathology of the liver biopsy showing normal hepatic cells with no evidence of atypia.

He had a liver biopsy which was negative for tumor, granuloma, inflammatory process, iron stains and fibrosis with preserved cyto-architecture (Figure 4).

The biopsy showed no sinusoidal dilation. Subsequently, he underwent exploratory laparotomy (with curative resection of the tail and body of the pancreas), partial gastrectomy, splenectomy, and retroperitoneal lymph node biopsy. During the surgical course, no evidence of splenic vein compression was found. Post-operatively, the patient was deemed diabetic and was placed on insulin therapy. Fortunately, he has been cancer-free since without any evidence of reoccurrence in one year.

## Discussion

PHT usually develops in the setting of liver cirrhosis, obstruction of hepatoportal flow, or parasitic infection. It is primarily defined on the basis of intrahepatic or extrahepatic causes. Extrahepatic causes are further divided into pre-hepatic or post-hepatic causes [4]. INCPH is defined as increased portal venous pressure in the absence of cirrhosis, splanchnic vein thrombosis or other causes of liver disease. The causes of INCPH can be divided into five categories [5].

A) Immunological disorders

B) Infections

C) Medications and toxins

D) Genetics

E) Thrombophilia

Gastric varices in the absence of EV are termed as IGV. They are also less common than EV. They occur in approximately 20% of patients with PHT. Splenic vein thrombosis is the most common causes of IGV [6]. The splenic vein running along the postero-superior aspect of the pancreas is vulnerable to compression or infiltration by adjacent pancreatic lesions. In the presence of PHT, there is shunting of blood from the splenic vein through the short gastric vein which gives rise to IGV. According to the classification of Sarin, et al., IGV occurring in the absence of EV have been classified into two groups [7]:

(1) Type 1 IGV (IGV1): varices located in the fundus

(2) Type 2 IGV (IGV2): ectopic varices in the antrum, corpus, and pylorus

Many studies have examined patients with IGV retrospectively and prospectively. Madsen and colleagues reviewed 209 patients with isolated splenic vein obstruction. Sixty-three percent of the cases were caused by pancreatitis, whereas pancreatic neoplasm contributed to 18% of the cases [5, 7]. Sutton and Madsen [8], in comprehensive reviews of English language publications from 1900-69 and 1969-84, respectively, identified only 263 cases. Almost half the cases were secondary to pancreatitis or its sequelae, notably pseudo-cyst formation. Only 36 cases were attributable to adenocarcinoma of the pancreas, the vast majority of which were not operable [3]. Levin and team published a study of 14 patients with IGV. Seven patients had evidence of PHT while rest of them had evidence of splenic vein thrombosis [9]. In the case series and literature review described by Thompson, numerous less common pathologies were reported, which included pancreatic pseudo-cyst, benign and malignant pancreatic neoplasms, iatrogenic splenic injury, wandering or ectopic spleen, infiltration by colonic tumor, spontaneous splenic vein thrombosis, and peri-renal abscess [10].

We did an extensive literature search to find case reports of gastric varices without evidence of splenic vein thrombosis; however, we did not come across any case report with a patent splenic vein causing IGV. Our patient’s work-up showed normal splenic venous flow. Intra-operatively, there was no evidence of compression or infiltration of the vein.

In our patient, the cause of PHT was not identified. The liver biopsy ruled out infiltrative, cirrhotic, or thrombotic process. Pancreatic cancers are known causes of splenic vein thrombosis, but in our case, venous ultrasound and surgical exploration did not reveal any thrombosis or compression of the portal venous system.

## Conclusions

Our patient was unique as there were gastric varices without splenic vein thrombosis and PHT without an identifiable cause and clinical manifestation. One should be vigilant about gastric varies on EGD in a patient with hematemesis even in the absence of splenic vein thrombosis. The EGD finding of gastric varies should prompt work-up for portal hypertension. All the common causes of portal hypertension should be ruled out before attributing the diagnosis of idiopathic portal hypertension.
